# Relational Databases for Behavior Science

**DOI:** 10.1007/s40614-025-00486-w

**Published:** 2025-12-02

**Authors:** Paul L. Soto

**Affiliations:** 1 Department of Psychology, Louisiana State University, 236 Audubon Hall, Baton Rouge, LA 70803 USA; 2 Pennington Biomedical Research Center, Baton Rouge, LA USA

**Keywords:** Relational database, Data collection, Data management, Spreadsheet, Data integrity, Reproducibility, Behavior

## Abstract

Data collection and analysis are central to scientific research, including in applied and basic behavior analysis. A substantial amount of attention has been given to how to rigorously collect and analyze data. Less attention has been paid to storing and maintaining research data, which becomes a critical step in the data analysis pipeline as the complexity and amount of data increase. Relational databases provide an efficient, reliable, and flexible method to store, maintain, and explore behavioral research data. The current article argues for the utility of relational databases in behavioral research, presents a brief introduction to relational databases, and uses some real-world examples to illustrate how relational databases have been used by the author and colleagues. Adopting relational databases to store and maintain research data would improve data integrity, facilitate data sharing between researchers, and contribute to transparency and reproducibility of analyses.

Data collection, storage, and analysis are key components of scientific research. Within behavioral science, researchers have many options for how they collect data, from written records to software-based data collection systems such as Med-PC (Med Associates Inc., Fairfax, VT), Multi-Option Observation System for Experimental Studies (MOOSES; Vanderbilt Kennedy Center), and Countee (Peic-Gavran & Hernandez Eslava, 2020). Compared to the attention given to data collection and analysis procedures, less attention has been given to the storage and maintenance of research data. Software-based data collection systems often generate a separate “flat” (so-called because all data are stored in a simple, two-dimensional structure of columns and rows) file (e.g., .csv or .txt) for each experimental session. Researchers can then manually input data into a spreadsheet by typing numbers stored in the flat files into the spreadsheet or use software or custom code to transfer data from the individual files into a program for analysis, such as a spreadsheet, graphing, or statistical analysis program. A common choice is a spreadsheet program such as Microsoft Excel. When the amount of data generated is small and the data organization relatively simple, a flat file-to-spreadsheet data pipeline can be a reasonable approach. However, as the number of files and the amount and complexity of the data increase, such a data analysis pipeline is vulnerable to many problems that compromise data integrity and reproducibility of analyses. For example, files may be inadvertently moved, leading to incomplete experimental data or irreproducible analyses. Alternatively, a data pipeline that uses a relational database for data storage and maintenance can address many of the issues that arise with a data pipeline that uses spreadsheets for storing and maintaining data. The current article provides an overview of the benefits of relational databases, how these benefits are achieved, and some real-world examples. For researchers interested in adopting a relational database approach, links to the author’s OSF projects containing databases configured for data files created by Med-PC software, MOOSES software, and Countee software are provided.

## Why to Use a Relational Database

Valid conclusions rest upon accurate data. Relational databases are useful for storing and maintaining research data because, if properly designed, they maintain data accuracy through the enforcement of rules called integrity constraints, that will be discussed below. In addition, relational databases are designed to efficiently store large amounts of data. For example, even a Microsoft Access database, which is a small-scale personal database, can store many millions of rows, whereas Microsoft Excel has a current limit of 1,048,576 rows. Relational databases also provide flexible methods for selecting, filtering, and summarizing data, which facilitates data exploration. Last, because queries for viewing, filtering, and summarizing data generate results on demand based on the underlying table data, reproducibility of analyses is possible by simply re-running the queries, and the addition of new data to the underlying tables is reflected in the query results when the query is executed. Next, the article provides a brief overview of when a relational database is appropriate for data storage and maintenance and what a relational database is followed by a discussion of the benefits of relational databases with a comparison to spreadsheet capabilities.

## When to Use a Relational Database

Relational databases are appropriate for storing and managing structured data when data accuracy and integrity are important. Structured data are data that can be stored in tables with predefined fields and rows that have a predictable and well-defined structure. Data collected by applied and basic behavior analytic researchers would typically fit well into a structured data scheme. For example, when conducting a single-case experimental design with individual participants or animal subjects, response events are often recorded in time within experimental sessions. Thus, the data can be stored in a table with a column for the time of the event and a column for the type of event (lever press, out-of-seat, pellet delivery, praise delivery, etc.). Similarly, structured data collected from group design studies would also be appropriate for a relational database.

Although summary data from an individual participant or animal subject in a single-case experimental design study might be easily managed in a spreadsheet, as the number of cases and recorded events grows, a relational database can become quite useful. For example, an operant experiment with nonhuman animals recording every response, reinforcer, and stimulus change event and their time of occurrence can easily lead to hundreds or thousands of events. Storing the results of such a study in a relational database would enable researchers to easily explore and summarize the results. Further, even though it may seem pointless to load data from a single participant or exploratory study into a database, over time, as the amount of data collected grows due to the addition of studies or participants or both, having all the data in a single database allows for easy analysis of the data across participants and studies. As an example, there is growing interest in combining data across studies involving small numbers of participants, combining data from individual clinical cases, or conducting prospective studies with large numbers of participants to assess the generality of the effects of applied behavior analytic interventions using single-case experimental designs (see Hagopian, 2020). Although data for an individual participant or client might be easily stored in a spreadsheet, storing the results from all participants or clients in a relational database would provide a useful single source of information for investigating questions such as for whom and under what conditions interventions are effective (see Hagopian, 2020, for a discussion of combining data across participants and clients).

In some cases, data may be unstructured and would require a different data storage approach. For example, image or video data (e.g., video or audio recordings of experimental sessions) would not be well-suited for storage in a relational database. Other situations in which relational databases may not be favored are situations involving massive amounts of data (e.g., Amazon) or situations in which the structure of the data is extremely complex and interconnected (e.g., a graph database; see Srivastava et al., 2023). The current article is targeted at behavioral researchers using a data pipeline involving creation of data files from experimental sessions that are then analyzed in spreadsheet-style programs such as Excel. Such data are structured and would be well-suited for storage in a relational database. Further, as the use of a relational database as a storage system for experimental data proceeds, a researcher could then begin to ask questions about the variables, such as characteristics of the subject or participant, that might moderate the effects of the manipulated variables.

## Overview of Relational Databases

A relational database is an electronic system for storing data in one or more tables. Tables are composed of rows and columns, and each column represents a variable or attribute, and each row represents an instance of data. For example, a table for storing participant information might have separate columns for the participant’s first name, last name, and date of birth. Each row in the table would represent the information for a specific participant. Tables in a database can be linked together using columns that they have in common (details provided below). Relational database systems range from small-scale (e.g., Microsoft Access) to large-scale (e.g., Microsoft SQL Server and Oracle) and from free (MySQL, PostgreSQL, and SQLite) to subscription-based (Access, SQL Server, Oracle). Large-scale systems are heavily used in the private sector. For example, businesses in areas such as telecommunications and health care utilize relational databases for the storage of customer and patient data, respectively. Although the amount of data that can be handled and the sophistication of tools available differ between small- and large-scale systems, the core design principles remain the same.

Data in a relational database can be managed (inserted, updated, and deleted) using Structured Query Language (SQL). SQL is a programming language, based on set theoretic logic, for relational databases. Data can also be viewed using SQL, and such queries can include aggregation operations, such as averaging, summing, and counting. Thus, SQL is the language for storing, managing (updating and deleting), and viewing data in a relational database.

## Benefits of Relational Databases

### Maintain Data Accuracy

#### Entity Integrity Constraints

**Fig. 1:**
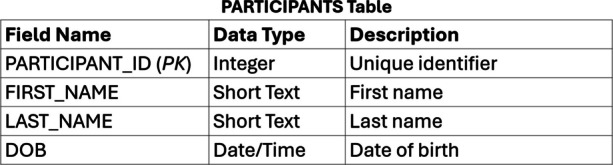
Table design for a table used to store information about participants in a study. *Note*. The table has four columns—a unique identifier for each participant, the first name, last name, and date of birth (DOB) of each participant. Each column has a defined data type preventing storage of other data types inadvertently. “*PK*” indicates the field is the primary key for the table

The primary goal of any storage system for research data is to maintain accurate data. A well-designed relational database maintains accurate data by enforcing rules, called integrity constraints, on what data can be stored in its tables. Integrity constraints are defined by the person who designs the database. There are two general types of rules that can be created by the database designer. The first type of rule, called an entity integrity constraint, is applied to the columns of the tables by specifying the column’s data type (e.g., integer, single, character; Figure 1). Data of another type than the defined data type cannot be stored in the column. For example, attempting to enter “2o” instead of 20 into the PARTICIPANT_ID column of the PARTICIPANTS table in Figure 1 would generate an error. Further, columns can be defined with allowable values to further restrict what values can be stored. For example, an integer type column could be specified to only allow values 1–10. Attempts to enter values outside the allowable range would generate an error. Finally, columns can include a NOT NULL constraint, which means that a value must be supplied for that column or a record cannot be added to the database. Via these mechanisms, the risk of incorrect data insertions or updates are reduced.

Spreadsheet programs such as Microsoft Excel also enable users to enforce data types and allowable value constraints. However, spreadsheet validation rules can be cumbersome to apply because they must be applied to specific cells and can easily be overwritten or lost if cells are moved or deleted. Spreadsheet programs do not have the capability to require that a cell contain a value, likely because all cells start blank (NULL) whereas in a database table, rows are inserted when data are added and rows do not exist otherwise. In addition, Excel’s autocorrect feature can introduce inaccuracies into spreadsheet cells. For example, Excel’s autocorrect feature has been repeatedly reported to cause issues with gene names that Excel interprets as dates (Abeysooriya et al., 2021; Zeeberg et al., 2004; Ziemann et al., 2016).

#### Referential Integrity Constraints

The second type of rule for maintaining data accuracy that can be enforced in a relational database, called a referential integrity constraint, is a rule that limits what can be stored in a column or table based on defined primary-foreign key relations. A primary key is a column (or group of columns) that provides a unique identifier for each record (row) in a table. For example, in the table shown in Figure 1, the PARTICIPANT_ID field is the primary key, denoted as “*PK*,” for the table and its value for any participant must be unique and cannot be null. A foreign key is a column (or group of columns) in a table that serves as a primary key in another table, allowing the user to link the data in the table containing the foreign key column to the table that uses the column as a primary key. For example, the unique participant identifier could appear with each session result in a separate table and could be used to link session results for each participant to the participant details. Referential integrity requires that, for a defined primary-foreign key relationship, the value in the foreign key column in a table must match one of the values in the corresponding primary key column. For example, suppose a database has two tables—one, called PARTICIPANTS, that contains information about participants such as name and date of birth, along with a primary key column, called PARTICIPANT_ID, providing a unique identifier for each participant, and another table, called SESSIONS, with data on observed behavior on each of multiple experimental sessions and a foreign key column called PARTICIPANT_ID. A referential integrity rule requires that any participant identifier value that appears in the PARTICIPANT_ID column (foreign key) of the SESSIONS table must be present in the PARTICIPANT_ID column (primary key) of the PARTICIPANTS table. An attempt to insert data into the SESSIONS table using a participant identifier not present in the PARTICIPANT_ID column of the PARTICIPANTS table would generate an error.

**Fig. 2:**
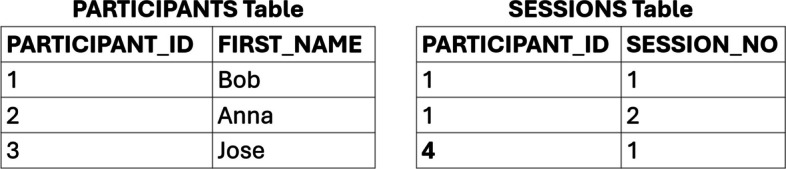
Violation of referential integrity

Referential integrity rules prevent orphaned records—rows of data in a table that do not have a primary key match in another table. For example, consider the two tables in Figure 2. The PARTICIPANTS table contains three entries, with unique identifiers for the participants of 1, 2, and 3. The SESSIONS table contains three entries—two sessions for the participant identifier 1 and one session for a participant identifier of 4, for which there is no corresponding entry in the PARTICIPANTS table. Referential integrity rules would prevent the insertion of the row in the SESSIONS table for which the PARTICIPANT_ID column is 4 because that value does not appear in the primary key column PARTICIPANT_ID in the PARTICIPANTS table. In such a situation, either the foreign key value is inaccurate, or the foreign key value needs to be added to the primary key table. By defining referential integrity rules, consistency and accuracy are maintained and enforced.

Spreadsheets do not have the capability to define and enforce referential integrity constraints. Data from different spreadsheets might be combined using formulas or loaded into another program, such as Python or R, and combined there, but no mechanism exists to ensure that data cannot be added in one spreadsheet if it would introduce inconsistencies between spreadsheets. For example, consider a situation where a researcher decided to store participant information, such as sex, age, and date of birth, in one spreadsheet and experimental session results in another spreadsheet. If they then wanted to combine participant information with experimental session results, the researcher would need to (1) use formulas to display data from one spreadsheet in the other spreadsheet or (2) join the data from the two spreadsheets in another program such as Python or R. But, in either case, there would be no way to ensure consistency between the two spreadsheets. Or a researcher might wish to store all data together in one spreadsheet, introducing substantial redundancies and risk of inaccuracies if updates or deletions were needed (see the Minimize Data Redundancy section, below, for more on this topic).

#### ACID Properties

Relational databases maintain data accuracy by ensuring that transactions, a group of related changes, adhere to four properties—Atomicity, Consistency, Isolation, and Durability (ACID). Atomicity refers to the property that a group of changes that occur within a transaction are all successful or the entire transaction fails. For example, in a transaction involving inserting data from an experimental session into a table and inserting related participant data into another table, if either insert failed for any reason, neither insert would succeed, thereby preventing the creation of inconsistencies between the tables. Consistency refers to the property that changes must move the database from one valid state to another. Thus, if a referential integrity rule or other rule prevented the insertion of data in one table as part of a group of changes within a transaction, the entire transaction would fail. Isolation refers to the property that changes to the same data are separated from each other. For example, if two users attempted to change the same data, the first transaction would need to complete before the next transaction could begin. Last, durability refers to the principle that successful transactions produce durable changes in the database, meaning that successful transactions are written to the computer’s disk drive and are not lost if a computer fails for some reason, such as a power outage.

### Minimize Data Redundancy

Efficiently designed data management systems (the database and integrity rules) minimize data redundancy. Data redundancy refers to the storage of the same information in multiple locations. Redundancy is undesirable for two reasons. First, redundancy unnecessarily increases the size of data storage files for obvious reasons. Second, redundancy hinders data accuracy by complicating maintenance operations, such as updates and deletes, and undermines referential integrity constraints. For example, if a participant’s information is stored in multiple tables, any updates to that information must successfully update all locations, or inconsistencies will be introduced. Unnecessary redundancy undermines referential integrity because enforcement of referential integrity constraints can only be applied to primary key—foreign key relationships. For example, if the participant’s unique identifier, name, and date of birth information were stored in the same table containing the experimental results, there would be no way to enforce referential integrity constraints on the name and date of birth information (i.e., to ensure consistency across rows for the same participant). Thus, maintaining accurate and consistent data requires that data redundancy be minimized, which well-designed relational databases do.

Relational database design principles are focused on reducing redundancy. Normalization refers to the rules and guidelines for designing tables and defining table relationships that reduce redundancies and thereby simplify data maintenance via the enforcement of the data validation and integrity constraints discussed above. The degree to which a database table is normalized is characterized by whether the table meets certain criteria called normal forms. There are six normal forms (Harrington, 2009), but only the first three will be discussed here, as these are the most straightforward.

**Fig. 3:**
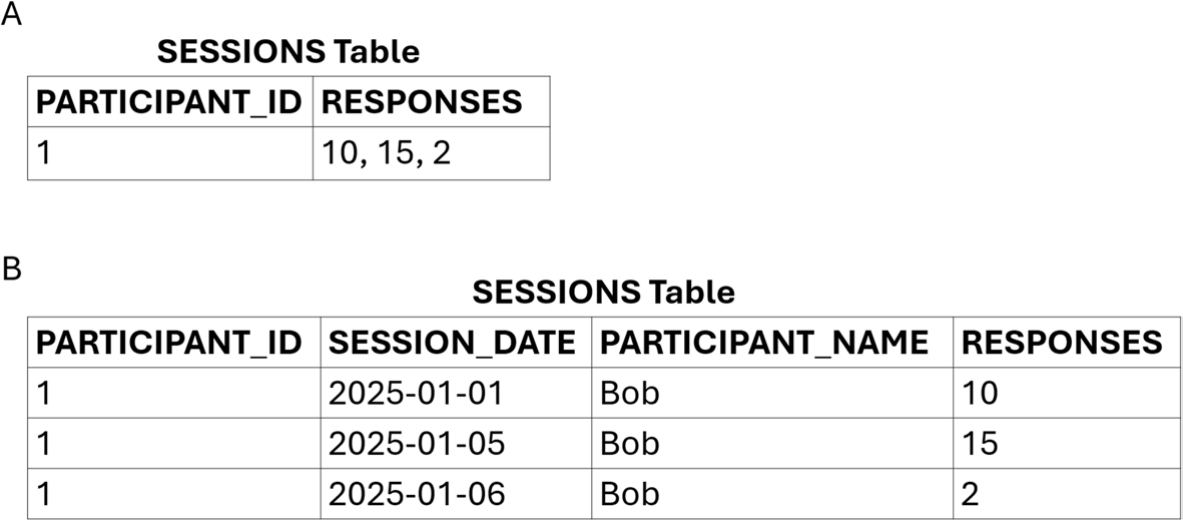
Violation of first (**A**) and second (**B**) normal forms

A table is said to be in first normal form if each column contains atomic values (a single value that cannot be broken down into meaningful components) and each row has a unique primary key. To illustrate this concept, consider the table in Figure 3A. A candidate primary key for the table would be the PARTICIPANT_ID column, assuming each participant has a unique ID and only one row exists for each participant. However, the table violates first normal form because there are three values (perhaps from three different sessions) recorded in the RESPONSES field of the table row. In that example, the RESPONSES field does not contain an atomic value, which would lead to various problems (e.g., no method to update or correct information about one of the three values, no method to select a particular session’s result for analysis). The solution to this violation of the first normal form would be to add a column that separates the three values into rows. For example, a column to record the session date, named SESSION_DATE, could be added to the table, resulting in three rows, one for each of the values shown in the RESPONSES column, and the primary key would be a composite of the PARTICIPANT_ID and the SESSION_DATE column (see Figure 3B).

A table with a composite primary key (a primary key that is comprised of two or more columns) is said to be in second normal form if the table meets the criteria for first normal form and all nonkey attributes (i.e., columns that are not part of the primary key) are fully dependent on the entire primary key (i.e., there are no partial dependencies involving a column that is part of the primary key and any nonkey columns). The table shown in Figure 3B has a primary key that is a composite of the PARTICIPANT_ID and SESSION_DATE columns but violates second normal form because the PARTICIPANT_NAME column depends only on the PARTICIPANT_ID (it does not depend on the SESSION_DATE column). The solution to this violation of second normal form would be to remove the PARTICIPANT_NAME column and to create a separate table to store participant information (e.g., the PARTICIPANTS table shown in Figure 2).

Last, a table is said to be in third normal form if the table meets the criteria for second normal form and there are no transitive dependencies, which occur when a non-key attribute depends on another non-key attribute. As an example, consider a table containing information about employees of an international company. In addition to a unique identifier for each employee and their name, the table includes residential information such as region and country. Assuming that regions are unique to a country, including both the region and country as columns would technically violate third normal form because of the dependency between region and country. A solution to this violation would be to remove the country column from the table and create a separate table to designate the country of each region. Using a separate table to store the region and country information would simplify maintenance because an update to the country name of a given region would only need to be done once in the region/country table, rather than for every employee that resides in that region.

Spreadsheets are not well-designed to reduce redundancy because they are not well-designed to handle one-to-many relationships, particularly multiple one-to-many relationships (Dilling, 2020). For example, in an operant study with nonhuman animals, a researcher will likely collect data in many experimental sessions for each subject. Thus, for each subject, there may be many sessions, which means a one-to-many relationship between subjects and sessions. Further, for each session, there may be many events, such as the time of each lever press or nose poke, which means a one-to-many relationship between sessions and events. When data involve multiple one-to-many relationships, spreadsheet approaches inevitably introduce profound redundancies and data maintenance challenges.

For example, consider a recent example from the author’s own research. Twenty-four mice underwent daily operant-conditioning sessions over a period of ~8 months. Across mice, the number of sessions was 148–149, comprising between 13,692 and 87,168 within-session events per mouse, for a total of 1,045,309 total recorded events. With a current row number limit of 1,048,576 for .xlsx files, we could store all the operant data alongside information about each mouse in a single spreadsheet, but we would introduce profound redundancies, which increase storage and maintenance issues. For example, suppose for subject-level information, we wanted to know the date of arrival, date of birth, date of death, strain, and sex of each animal. Storing each of these pieces of information alongside each recorded event would require repeating each piece of subject information tens of thousands of times (once per each event recorded per animal). Storing session-level information such as the start datetime and end datetime of the session alongside each event would require repeating the session information thousands of times (once per event within a session). Update operations to one field, such as the date of death, would require updating tens of thousands of cells. Even if we decided to accept the redundancies and update issues, a spreadsheet with that many rows would likely be slow, the program might crash, and we could not add (much) more data, even if it were deemed experimentally desirable. In contrast, adding another million or so rows to a database would be trivial.

As an alternative, to avoid unnecessary repetition in the single spreadsheet approach, a researcher might store subject-level information in one spreadsheet, session-level information in another sheet, and events within sessions in a third sheet, either within the same file or in separate files. However, this multiple spreadsheet approach would introduce difficulties in pulling together data for analysis because, as described above, spreadsheets are not designed to link information together from different tables. For example, if a researcher wanted to calculate the age of the subject on each session, there would be no simple way to join the information in the subject- and session-level spreadsheets. Or, if a researcher wanted to conduct a calculation on the event-level data, such as the average response rate in male versus female animals, there would be no simple way to use the information in the subject-level spreadsheet to define the aggregation on the event-level spreadsheet. A workaround in Excel, as an example, might be to use formulas to pull information from one spreadsheet into another, but this would then produce the unnecessary repetition that the researcher was attempting to avoid in the first place!

To reduce unnecessary data redundancy, a determined spreadsheet user might conclude that it is unnecessary to store event-level data from their experiment. Perhaps the researcher only cares about three variables in each session, such as responses, reinforcers, and session duration. For 24 subjects with 148 sessions each, this amounts to 3,552 rows in a spreadsheet, a not unwieldy number of rows. However, such an approach to reducing data redundancy would severely limit the researcher’s ability to explore their data and ask other questions. The ability to conduct fine-grained analyses of our data increases our data intimacy and may yield new insights into the variables governing behavior (for a discussion of data intimacy in applied behavior analysis, see Fahmie & Hanley, 2008). For example, imagine that a researcher wanted to calculate the latency to the first response in each session or the time in the session at which subjects stopped responding, but the researcher had only stored summary-level data in a spreadsheet. To answer such questions, the researcher would need to return to the original flat files and get the data, enter the data into the spreadsheet, and conduct the new analysis. Such an approach would be tedious and time-consuming, whereas the results could be obtained within minutes if the event-level data had all been stored in a relational database.

### Query and Summarize Data

A useful data storage system must provide a flexible method for querying and summarizing stored data. Nearly all relational databases use SQL to view and summarize data. SQL statements can pull data from one or more tables using joins between keys that link the tables (examples provided in the Some Real-World Examples section). SQL statements can include filters for limiting what rows are included and aggregation functions for performing mathematical calculations on specified fields. For example, in a database containing participant and reaction time results tables, an SQL statement could pull desired participant and reaction time information together in one result set, along with calculating the average, maximum, and minimum reaction time. It is important to note that SQL statements of this kind, in contrast to the insert, update, and delete statements used to manage a database, do not alter the underlying data. Thus, the researcher can flexibly pull data from one or more tables, sort, filter, and perform various mathematical operations such as counting, averaging, and finding the minimum and maximum values of a field without affecting the underlying data. Further, SQL statements can be generated and executed from scripts written in programming languages such as R or Python allowing programmatic data access, manipulation, and analysis.

It is critical to remember that data manipulation in spreadsheets is error prone. For example, copy/paste errors such as copying fewer rows or columns than intended or pasting into unintended cells are easy to make. Likewise, data sorting errors such as sorting some columns causing misaligned data are easy to make. As a well-known example, the failure to reproduce an analysis of cancer genotypes in chemotherapy selection was partially attributed to an “off-by-one” sorting error in Excel in which the original researchers sorted some rows and not others (Hutson, 2010). In addition, formulas in spreadsheets for performing aggregation operations are susceptible to errors. For example, formulas may accidentally include incorrect cell references and when adding new rows to a spreadsheet data table, relevant formulas must be updated or the calculation will be incorrect or may simply return an error message.

### Support Reproducibility

There is growing recognition that data analyses should be reproducible (Nosek et al., 2022). By providing on-demand generation of summary analyses that do not alter the underlying data, SQL provides results that can easily be reproduced because the queries can be re-run at any time. Further, the SQL can be scrutinized to understand how the results are generated. It is important to note that SQL cannot be used for advanced statistical analyses. Nor do relational databases typically contain the capacity to produce data visualizations. For advanced analyses and data visualizations, researchers will need other software, but the relational database will remain the central data storage and management location. For advanced analyses and data visualizations, data in the database can be accessed using various approaches. As a simple approach, researchers can export summary results produced with SQL (written by the researcher or created using a visual query builder) for import into and analysis in the R programming language (R Core Team, 2018) or the Python programming language. As an additional note, analyzing and visualizing data using code also facilitates reproducibility because all analysis steps can be documented in the code. Or researchers can directly interface with the database from programs such as R and Python to pull data from the database for analysis and visualization either by running queries against the database or simply pulling data from the tables and then combining the selected data in the programming environment.

Reproducibility of analyses is difficult when analyses are conducted in spreadsheets. Various operations that researchers might conduct to analyze data cannot be tracked. Data sorting, copy/paste, and cut/paste operations that may change underlying data are not documented when working in a spreadsheet. Thus, it may be difficult or impossible to recreate an analysis conducted using a spreadsheet program.

### Other Benefits

There are several other benefits to relational databases that will be briefly mentioned here. First, relational databases provide fast performance when querying and summarizing even large amounts of data. One of the ways this is achieved is with indices on table columns that allow SQL to operate quickly. Second, many relational databases provide multi-user access so that members of a research team can work on the database simultaneously. Third, some relational databases allow the user to implement sophisticated security controls over the data, such as user-based table-, column-, and row-level security and also to implement controls over what actions each user can take in the database. For example, in a large research team working with sensitive personal data, controls on the data could be implemented so that undergraduate research assistants would not have access to personally identifiable information whereas the principal investigator would have access to all the data. As another example, only selected users could be given rights to update or delete data whereas others would only be given rights to view data. The next section will discuss some examples of table designs for researchers who typically work with spreadsheets.

## Table Design Examples

**Fig. 4:**
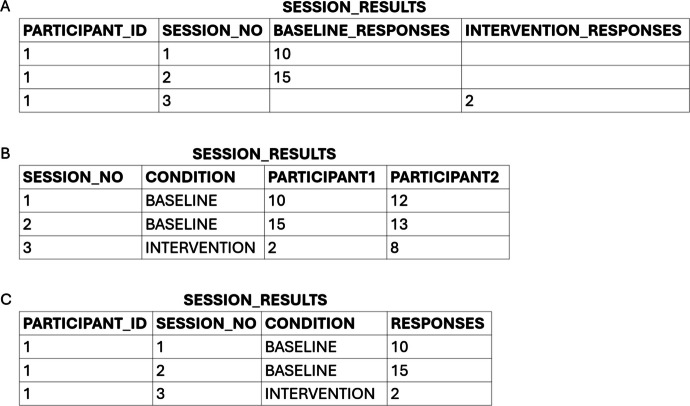
Spreadsheet layouts (**A** and **B**) versus normalized table format (**C**)

Sound database design involves normalizing tables, as described in the section Minimize Data Redundancy. To better understand the process of normalization, it may be helpful for researchers unfamiliar with relational databases to consider the problems associated with spreadsheet organization in contrast to table normalization. Data spreadsheets are often organized in ways that are intuitive to review but difficult to maintain without a high degree of manual process^1^. For example, consider Figure 4, which shows two problematic layouts (Figure 4A and B) and a normalized table design (Figure 4C). In Figure 4A, the number of responses observed in each experimental session is separated into two columns—one for sessions that occurred during a baseline condition (sessions 1 and 2) and one for sessions that occurred during an intervention condition (session 3). A layout such as the one shown in Figure 4A might be used because it allows the user to easily graph baseline and intervention data using different symbols in programs like Microsoft Excel, but it violates first normal form, poses management difficulties, and hinders analysis. It violates first normal form because it contains what is called a “repeating groups” structure in which the same data, response totals in the example, are stored in multiple columns, potentially introducing null values, leading to ambiguity about whether those fields represent sessions in which no responses occurred, but could have. It poses management difficulties because the table structure must be modified if an additional condition is conducted. Last, it hinders analysis by including data (i.e., the condition to which each session belongs) in the structure of the table (i.e., in the column names), preventing the user from flexibly selecting or aggregating data based on condition. A better design would be to modify the table to include columns for PARTICIPANT_ID, SESSION_NO, CONDITION, and RESPONSES as shown in Figure 4C.

As another example of a common spreadsheet layout, consider the layout shown in Figure 4B. In this example, each participant’s results are stored in a separate column. Such a design is problematic for the same reasons as the design shown in Figure 4A. The design violates first normal form for the same reason that the design in Figure 4A violates first normal form—it introduces repeating groups with associated null values. The design introduces management issues. For example, if the condition field does not apply to all participants, there is no way to differentiate the condition by participant. For example, as shown, sessions 1 and 2 were baseline condition sessions. If, however, session 3 was also a baseline session for participant 2 but not for participant 1, the design would not provide a method for recording this fact. Further, the design assumes a known and fixed number of participants (2 in this case). If another participant were enrolled in the study, the table design would need to be revised to add a new column. Finally, the design introduces difficulties if the researcher wants to summarize or analyze results based on participant characteristics (e.g., grade level, sex) because there is no straightforward way to select data based on participant characteristics. A better design would be the design shown in Figure 4C. By adding a PARTICIPANT_ID column, the researcher could record the condition information for each participant’s session separately and store the outcome variable of responses in a single column for efficient analysis, including analysis by participant characteristics, which would be available in a table storing participant information.

It is important to remember that the goal of normalization and database design is to simplify data maintenance (correction of errors or information changes via updates and/or deletions) and thereby improve data integrity. By normalizing the tables in a database, we ensure that each piece of information is stored in a single location and that there is no unnecessary repetition. We can then use SQL to flexibly join the data in our tables and to summarize our results. The next section briefly discusses the creation of a database and how tables can be populated with research data.

## Creating and Populating a Database

Assuming a researcher wishes to use a relational database to store and maintain their research data, the researcher would need to create the tables, set the primary keys for the tables, define the relationships between tables, and identify a method for loading data from flat files generated by data collection software into the database tables. Database creation steps depend on the specific application but can typically be done via SQL statements or through a graphical user interface (GUI). For example, in Microsoft Access, it is easy to use the GUI to create tables, define the columns with their data types, and specify the primary key for the table. Likewise, the default relationships (primary key to foreign key relationships) between the tables can be defined via GUI in Microsoft Access.

For loading data from data sources such as flat files, several commercial software packages allow the user to define the data pipeline involving reading the source data files, extracting relevant information, performing any transformations necessary to get the data in an appropriate form for loading into tables, and last, loading the extracted data into the database tables. These tools are called ETL tools, for Extract, Transform, and Load and they provide click-and-drag user interfaces for defining steps in the process of reading and extracting data from sources, transforming data into needed formats, and loading tables with the transformed data. Some are open-source and free, and others are available for purchase (e.g., Talend Open Studio, Qlik Technologies Inc., Pentaho Data Integration, Hitachi Ventara). In general, such tools would be unnecessary for an individual researcher’s needs. A low or no-cost alternative for an individual researcher is to write custom code to read data sources and use SQL insert and update statements to insert and update data, respectively. Code can be written in languages such as Python or Visual Basic for Applications, which is included in the small-scale Microsoft Access database application. As an alternative, for researchers not comfortable with coding, basic import features in Microsoft Access might suffice for certain types of files, although the import process would need to be conducted repeatedly, once for each file. For interested readers, examples of using VBA code within Microsoft Access to read source data files and load tables are discussed below and provided in the author’s Open Science Framework account.

## Some Real-World Examples

**Fig. 5:**
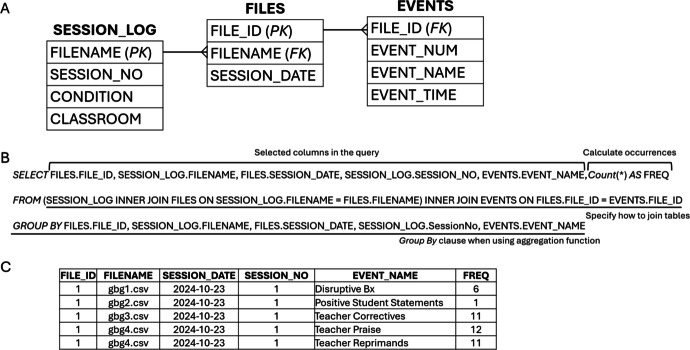
Tables and relationships in a database designed for storing data from GBG study in classrooms (**A**), SQL select statement for generating a summary of events within sessions (**B**), and sample output from SQL statement (**C**). *Note*. “FK” indicates the field is a foreign key that relates to the primary key another table

To illustrate the application of relational database technology to storage, maintenance, and summary of research data, two examples will be discussed. The first example is a relational database designed for data collected using the Countee application (Peic-Gavran & Hernandez Eslava, 2020). The study involved the implementation of the Good Behavior Game in school classrooms. Countee software was used to record events during the Good Behavior Game sessions and text files for each session were created by the application. Over the course of the study (unpublished), 172 files covering over 13,000 recorded events were produced. A database was created in Microsoft Access that used two principal tables—a table for storing information about the files and a table for storing events recorded in the files (Figure 5A). The FILES table included a column for the file identifier (FILE_ID), which served as the primary key. In addition, the filename (FILENAME) and the date of the session (SESSION_DATE) were recorded in the FILES table. The EVENTS table contained a column for the file identifier (FILE_ID) that served as a foreign key to the FILES table, a column that contained the number of each event within a session (EVENT_NUM), a column that contained the name of each event (EVENT_NAME), and a column that contained the time of occurrence of each event (EVENT_TIME). The EVENTS table used a composite primary key formed by the FILE_ID and EVENT_NUM. Finally, the database also contained a link to a Microsoft Excel file for recording attributes of each Countee data file that were not stored within the file (e.g., attributes such as the experimental condition and session number). During the course of the study, the Excel file was used because it was easier for students to maintain information about the sessions in Excel rather than maintaining the information in the database. Within the database, the linked Excel file was called SESSION_LOG and contained columns for the filename (FILENAME), the session number (SESSION_NO), the condition (CONDITION), and the classroom (CLASSROOM). The relationships between the tables are shown in Figure 5A.

During the study, SQL (Figure 5B) was used to select specific columns from each table and to count the number of occurrences of each event to generate a final output result (Figure 5C) for analysis and visualization. Similar approaches were used in other published applied behavior-analytic studies (Donaldson et al., 2015; Gomes et al., 2025). Template databases with code for loading files created with MOOSES or Countee software are available in the author’s Open Science Framework account (Soto, 2025a, 2025b).

**Fig. 6:**
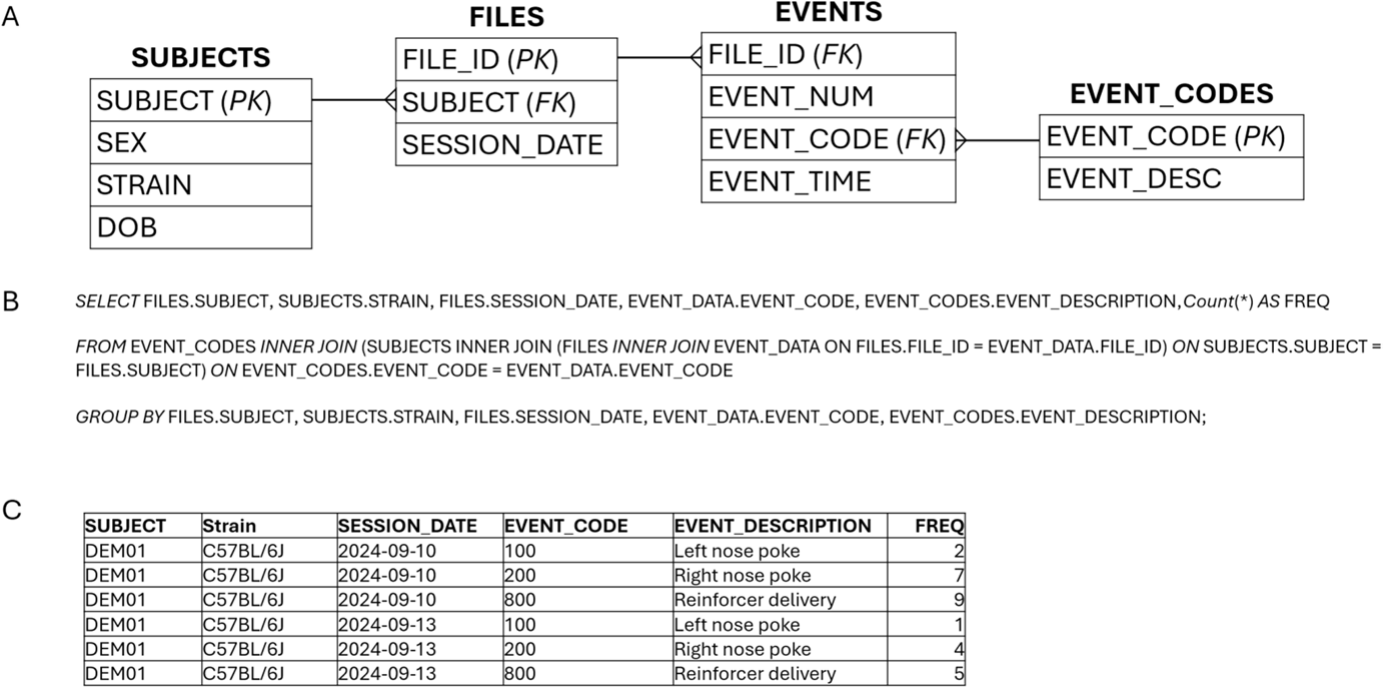
Tables and relationships in a database designed for storing operant session data (**A**), SQL select statement for generating a summary of events within sessions (**B**), and sample output from SQL statement (**C**)

The second example is a database created for data collected in a study of operant behavior in mice. Med-PC V software (Med Associates, Inc.) was configured to generate one text data file per subject per session. Over the course of the study, 3,564 individual text files covering 1,045,309 recorded events were generated. The database contains four key tables—a table for storing information about the text data files (FILES), a table for storing which events occurred and the time of each event that occurred within the experiment sessions (EVENTS), a table for storing information about the animal subjects (SUBJECTS), and a table for storing a description of each event that could occur in a session (EVENT_CODES). The FILES table contains a unique identifier for each file (FILE_ID) and file attribute columns (Figure 6A, not all attributes shown). The EVENTS table contains a FILE_ID column (foreign key to the files table) and a column to number each event that occurred in a session (EVENT_NUM; unique within each file ID), which together form a composite primary key for the events table. Also included in the EVENTS table is a column to record the event codes that identify events (EVENT_CODE; e.g., response on left response option, response on right option, reinforcer delivery) and a column for recording the time of occurrence of the event (EVENT_TIME). The SUBJECTS table contains the unique subject identifier (SUBJECT) and a series of subject attributes (sex of mouse [SEX], strain of mouse [STRAIN], date of birth [DOB]). Finally, the EVENT_CODES table provides a lookup for each EVENT_CODE to provide a full-text description (EVENT_DESCRIPTION).

For summary analysis, SQL was then used to join the tables and pull information from the SUBJECTS, FILES, EVENT_DATA, and EVENT_DESCRIPTION tables and to count the number of occurrences of each event code (Figure 6B), producing the output shown in Figure 6C (only a subset of rows shown). The resulting data were then exported to .csv file for import into R for statistical analysis and visualization. My colleagues and I have used this approach for the storage, maintenance, and preparation of data for analysis and visualization in a number of basic behavioral and behavioral pharmacology studies (e.g., DiMarco et al., 2023; Hiranita et al., 2025; Hiranita et al., 2009; Hiranita et al., 2013; Soto & Hiranita, 2020; Soto et al., 2023; Torres et al., 2022). A template database with code for reading data files created by Med Associates software (Med Associates, Inc.) is available in the author’s OSF account (Soto, 2025c, March 14).

## Challenges to Adoption

Adoption of a relational database management system will require the development of specialized skills on the part of at least some members of the research team. Up-front work will be required to design the overall data schema—the design of the tables, the primary key–foreign key relationships between tables, and the integrity constraints imposed on columns. The overall data schema can be defined using SQL and queries can be written in SQL, but most databases have graphical user interfaces that make these tasks easier. For example, Microsoft Access has a graphical user interface for defining relationships and a visual query builder. PostgreSQL has a tool called pgAdmin, which is an interface for creating and maintaining PostgreSQL databases and for creating queries. Microsoft SQL Server has Microsoft SQL Server Management Studio for creating and managing SQL Server databases and for creating queries. In addition, there are tools such as DBeaver, which can connect to various databases, and can make table creation, database management, and query creation much easier than writing SQL directly.

A researcher wishing to utilize a relational database system will need to make decisions that will entail various technical requirements. First, the researcher will need to decide whether to support multiple concurrent users. If the answer is yes, the database selected will need to support a server–client architecture and be installed on a server computer with network access configured. Further, user accounts will need to be set up or integrated with established institutional network accounts. Such technical requirements would typically require support from one’s institutional information technology (IT) department and would entail financial costs in terms of hardware and software, depending on the database selected (MySQL and PostgreSQL are free; Microsoft SQL Server must be purchased unless an institutional license exists). Multiple concurrent user access is not supported by some small-scale databases, such as Microsoft Access and SQLite, and providing multiple concurrent user access therefore requires the selection of a database that provides such access (e.g., MySQL, PostgreSQL, Microsoft SQL Server).

The researcher will also need to decide whether access to the data will need to be differentiated between users (e.g., restrictions on what data are available to each user). If the answer is yes, the researcher will need to select a database that supports the ability to impose restrictions based on user accounts or roles. Databases like MySQL, PostgreSQL, and Microsoft SQL Server offer such functionality and can be installed on a local machine (e.g., a lab computer that lab members log into with their user accounts) if concurrent access is not required (e.g., team members work on the local machine, one at a time) or on a server machine, if concurrent access is required.

Another technical requirement will involve the selection of a backup option. Small-scale databases such as Microsoft Access do not offer automated backup options and therefore require manual backup of the database file on a regular basis. Larger-scale databases offer various backup options that enable recovery of the database in case of failure. Configuring those options would require database-specific configuration by the researcher and/or assistance from the institutional IT department, particularly if the database management system is hosted on a server computer.

As noted above, larger-scale approaches such as configuring a server-client database on a server computer will require support from IT specialists. Or a researcher might choose to leverage existing institutional resources. Some universities participate in REDCap consortia that provide university researchers access to the REDCap system, which provides a browser-based interface to a relational MySQL database for storing and managing research data (Harris et al., 2019; Harris et al., 2009), thus removing the burden of technical requirements from the researcher. Another approach to adopting a larger-scale relational database system without incurring the burden of technical requirements is to use cloud-based services such as Amazon RDS, Azure SQL Database, and Google Cloud SQL, which charge based on usage. Such services offer benefits such as automatic backups, high availability, and granular restrictions based on user login. For an individual lab with low demand usage, such services can be affordable, and Azure SQL Database offers a free tier designed for learning and development that might be sufficient for small labs or projects. Cloud-based relational databases can also provide HIPAA-compliant access based on a shared responsibility model involving the provider and user. In the end, the user is responsible for ensuring the database is configured to align with HIPAA requirements.

In summary, the development and use of a large-scale database system providing concurrent user access can require substantial technical and possibly financial resources. Smaller-scale options are for researchers to use available hardware (shared lab computers) to install open-source database software and avoid concurrent use. For a researcher moving from spreadsheet-based data management and analysis, moving to a small-scale database such as Microsoft Access or a locally installed platform such as MySQL or PostgreSQL would provide the benefits described in this article without the most burdensome technical and financial requirements. If research needs begin to exceed what can be handled by such small-scale approaches, data could then easily be migrated to a cloud-based service.

## Conclusions

Relational databases provide an efficient method for storing and managing behavioral research data. The benefits of adopting such an approach include the ability to ensure data accuracy and to facilitate data analysis by allowing the researcher to select, combine, and summarize large quantities of data and to combine fine-grained analyses of their data. Further, by simplifying and standardizing data storage, relational databases facilitate combining data across studies and sharing of data in support of open science efforts, ultimately benefitting the reliability of behavioral science (Gilroy & Kaplan, 2019; McKiernan et al., 2016; Nosek et al., 2022; Tincani et al., 2024). For interested researchers, there are many resources on the internet for learning SQL (Khan Academy, n.d.; W3Schools, n.d.), as well as books on database design that are available for purchase or freely available (e.g., Harrington, 2009; Watt & Eng, 2014). In addition, artificial intelligence systems such as ChatGPT can be useful for design and SQL suggestions. Adoption of relational database technology for behavioral research would improve research data integrity and thereby improve transparency and reproducibility of research in behavioral science.

## Data Availability

The manuscript does not report research findings and therefore no data are provided. Examples of the databases are provided in the author’s OSF (links available in references).

## Footnotes

1 Spreadsheets often include nondata features such as conditional formatting that are not discussed here but are problematic for analysis (e.g., no method for counting cells with the property “red”).
